# A Visit to Stockholm Hospitals
*Article I. appeared last week.


**Published:** 1915-02-06

**Authors:** 


### II.—A VISIT TO STOCKHOLM HOSPITALS.*

### By a Travelling R.M.O.

Another large hospital in Stockholm is the Serafina Lazaret. This institution is an important one and possesses an eminent staff of consultants. The buildings consist of large blocks which have seen a good many years' service. The medical wards partake of the old-fashioned character and mostly contain about fourteen beds, but are bright and airy; they open out on wide, well-lit corridors. The pride of this hospital is the magnificently equipped *x*-ray department. This is housed in a block by itself and is provided with the most modern apparatus for therapeutic and diagnostic work. The ingenuity with which the electrical connections are arranged surpasses anything to be seen in this country. Different portions of apparatus can be connected up by the working of levers in an adjoining room without any necessity for altering the wiring by hand. Any false connection is signalled by an alarm bell and by the shutting off of the current. The heavy blinds which convert the room into a pitch-dark chamber are simultaneously drawn by the working of electric motors. Such an equipment is rendered possible by the fact that the municipal purse is available. The director of this department is, of course, engaged in research work, and has the assistance of quite a modern office equipment for the keeping of records and sending out of clinical reports on hospital patients. In the director's room were to be seen filing cabinets, a typewriter, and, above all, a dictaphone! The professor in charge of the medical side of the hospital has his own private laboratory and office adjacent to the wards, where he has the help of a sister-secretary and one or two medical assistants. The shady grounds of this hospital, which is situated in a populous district, are rather limited in extent if viewed with German eyes, but what would not some of our London hospital committees give for equivalent space around their institutions, in which the patients could stroll or sit! The winter months, even in Sweden, provide many days on which out-door exercise can be taken by hospital patients.

### Sisters as Anæsthetists.

One more detail may be noticed in passing—namely, the frequency with which the administration of anæsthetics is entrusted to a sister instead of to one of the medical staff. This plan has been found to work exceedingly well. The sister by whom this work is performed soon becomes a real expert, and in the opinion of the surgeons is more patient, conscientious, and reliable than a qualified medical man would be, unless, indeed, he were exclusively in practice as an anæsthetist. Such specialists are much rarer apparently in Stockholm than in London. It certainly seems a good plan under the circumstances, for the unqualified female expert might well be a better administrator than the intermittent though qualified anæsthetist who has other work to devote himself to. But the principle is one which is not likely to find favour in England.

* Article I. appeared last week.

